# The association between sleep duration and excess body weight of the American adult population: a cross-sectional study of the national health and nutrition examination survey 2015–2016

**DOI:** 10.1186/s12889-021-10369-9

**Published:** 2021-02-11

**Authors:** Qing Li

**Affiliations:** School of Humanities and Social Sciences, Guangxi Medical University, No. 336 Wuming Street, Wuhua Road, Wuming District, Nanning, Guangxi 530100 P.R. China

**Keywords:** Sleep duration, Body mass index, Obesity, Overweight, NHANES

## Abstract

**Background:**

We intend to explore whether sleep duration is associated with overweight and obesity among the adult American population. Furthermore, we stratified the study population by age and sex in the subgroup analysis to investigate the potential disparities between adults and older adults, and men and women.

**Methods:**

In total, 2459 individuals from the 2015–2016 National Health and Nutrition Examination Survey cycle were included for analysis in this study. Sleep duration was assessed by the Sleep Disorders Questionnaire. Classification of the short-sleep, normal-sleep, and long-sleep group was based on the recommendation of the National Sleep Foundation. Bodyweight was measured during the physical examination. Multivariate logistic regression models were implemented.

**Results:**

We observed a significantly higher overweight incidence in the short-sleep group compared to the normal-sleep group (OR = 1.825, 95%CI: 1.251–2.661, *P* = 0.004). Short-sleep (OR = 1.832, 95%CI: 1.215–2.762, *P* = 0.007) duration and long-sleep duration (OR = 1.370, 95%CI: 1.043–1.800, *P* = 0.027) were associated with higher prevalence of obesity. When stratified by age, short-sleep also increased the overweight and obese incidence 1.951 and 1.475 times in the adult group. In the sex-stratified subgroup analysis, the short-sleep group showed 2.49 times higher overweight incidence among females. The prevalence of obesity was 2.59 times higher in the short-sleep group and 1.698 times higher in the long-sleep group in the female population.

**Conclusions:**

Sleep duration is associated with the occurrence of overweight and obesity, with sleep duration less than 7 h increase the overweight and obesity rate nearly 2 folds comparing to sleep 7–9 h.

## Background

Excess body weight remains one of the top health concerns due to its association with several chronic diseases, such as a higher risk of cardiovascular disease, insulin resistance, and hypertension [1]. Globally, approximately 1.9 billion (39%) adults were overweight, and 609 million (13%) adults were obese in 2015 according to the World Health Organization [2, 3]. In 2014, the estimated obesity rate in 20 European countries was 15.9% and the prevalence of obesity and overweight in China was 28.1% and 5.2% [4, 5]. However, the obesity incidence in the U.S. is much higher than the global obesity prevalence. In the United States, the national prevalence of adult obesity has increased from 30.5% in 1999–2000 to 42.4% in 2017–2018 and has been predicted to reach 48.9% by 2030 [6, 7], raising substantial concerns and attention regarding the prevention and management of overweight and obesity. Female adults as compared to male, 40–59 years old middle-aged adults as compared to other age groups, and non-Hispanic black as compared to other race groups indicate a higher prevalence of obesity [7].

The recommended sleep duration, according to the National Sleep Foundation, is 7 to 9 h for young adults and adults, and 7 to 8 h for older adults [8]. Notably, approximately 50% of Americans reported spending 7 to 9 h in bed, while 15% of Americans reported spending less than 7 h in bed on weeknights [9]. Short sleep duration is associated with decreased intake of dietary fiber and increased intake of carbohydrates, total sugar, total cholesterol, and total saturated fat [10], which leads to superfluous caloric intake and disrupts the balance between energy intake and energy expenditure.

Moreover, significant changes in the satiety regulatory hormones have been observed independently from BMI among participants with short sleep [11]. The decreased leptin and elevated ghrelin suggest a possible increase in appetite of the study participants, which may explain the excessive energy intake. Changes in appetite regulatory hormones underly the potential role of sleep duration in preceding weight gain.

Therefore, this study aimed to examine the relationship between sleep duration and excessive adiposity of the adult population in the United States.

## Methods

### Study design

In this population-based cross-sectional study, data from the National Health and Nutrition Examination Survey (NHANES) 2015–2016 cycle was analyzed. NHANES was a continuous nation-wide program that collected population-based data on a 2-year basis. NHANES program consisted of two parts, an in-home interview and a physical examination in the Mobile Examination Center (MEC). In each 2-year survey cycle, NHANES collected approximately 10,000 nationally representative stratified sample across all ages. This study analyzed publicly available data downloaded from the NHANES official website and was exempt from future ethics review board approval of the Guangxi Medical Hospital.

In total, 9971 people participated in the NHANES 2015–2016. Since this study aimed to investigate the relationship between sleep duration and overweight and obesity in the adult American population, participants with a BMI less than 18.5 (*n* = 1731), and participants less than 18 years old (*n* = 2341) were excluded. Individuals with missing information in age (*n* = 423), sex (*n* = 666), BMI (*n* = 330), sleep duration (*n* = 651), marital status (*n* = 262), education level (*n* = 487), and daytime sleepiness (*n* = 585) were also excluded. A total of 2495 participants were eligible for analyses in this study.

### Sleep duration assessment

In this study, sleep duration was assessed based on the data of the NHANES Sleep Disorders Questionnaire (SLQ). The questionnaire was administered during the interview, the full procedure of which was available on the NHANES official website (https://wwwn.cdc.gov/nchs/nhanes/continuousnhanes/questionnaires.aspx? BeginYear = 2015) [12]. Consisting of six questions, the SLQ collected information regarding the usual sleep and wake time, sleep hours, snore frequency, snort and apnea frequency, insomnia, and daytime sleepiness. Question SLQ012 asked, “How much sleep {do you/does SP} usually get at night on weekdays or workdays?” Participants responded the time they fell asleep during their main sleeping period, including both short and long sleep time if the individual woke up periodically. The reported duration was rounded to the nearest half-hour. The short-sleep group included participants who reported sleeping less than 7 h, while the long-sleep group contained individuals who reported sleeping more than 9 h. The normal-sleep group, including participants who reported sleeping 7–9 h, was defined based on the recommendation of the National Sleep Foundation [8]. In the subgroup analysis stratified by age, individuals with a self-reported sleep duration at 7–8 h were categorized as the normal-sleep group for older adults aged 65 years or older, as recommended by the National Sleep Foundation [8].

### Bodyweight measure

Anthropometry examination was performed in the MEC by medical professionals. The full procedure, including the protocols, equipment, data entry, and quality control, was described at https://wwwn.cdc.gov/nchs/nhanes/ContinuousNhanes/Manuals.aspx?BeginYear=2015 [13]. Bodyweight was measured using a calibrated digital weight scale. Participants changed to a standard MEC exanimation gown with only underpants beneath it before weighting. After clothes change, the participants were directed to stand in the center of the digital scale with hands at sides and eyes looking straight forward. The weight was collected in kilograms and shared with the participants if inquired by the participants. Standing height was measured using a stadiometer with a fixed vertical backboard and adjustable head piece. The participants were barefoot and were asked to remove all hair ornaments. After being guided to the stadiometer, the participants were asked to stand straight with their head, shoulder blades, buttocks, and heels touching the vertical backboard. Corresponding BMI (kg/m^2^) was calculated and rounded to 1 decimal place. Weight status was determined using BMI, which was available in the NHANES examination database as variable “BMXBMI.” For the purpose of this study, BMI was categorized into a normal weight group (BMI 18.5–24.9 kg/m^2^), overweight group (BMI 24.9–30 kg/m^2^), and obese group (BMI ≥30 kg/m^2^) based on the WHO standard [3].

### Potential covariates

Socioeconomic status, education level, race, family composition, and marital status were associated with obesity and sleep [14–19], and were therefore analyzed as potential covariates. Poverty Income Ratio (PIR), which reflected the economic status of the study participants, were categorized to < 1.56, 3.61–4.56, and ≥ 4.56 based on the tertiles. Since the recommended sleep duration was different between adults and older adults [8], subgroup analysis stratified by age was performed. Based on the indicator definition of the Center for Disease Control and Prevention [20], participants aged 65 years or older were classified as the elder group, while participants aged younger than 65 were categorized as the adult group. Group definition of other variables was based on the categorization of the NHANES survey.

### Statistical analysis

The distribution of variables was analyzed using the Shapiro normality test. Normally distributed continuous variables were presented as mean  ±  standard deviation (Mean ± SD), while abnormally distributed continuous variables were divided into quartiles (Q). Categorical variables were expressed as frequencies and percent distributions (N%). Comparisons of demographic characteristics and potential covariates between the normal BMI group and overweight and obese groups were conducted using independent sample t-test and Mann-Whitney U test for continuous variables, and Fisher’s exact test and Pearson’s chi-square test for categorical variables.

The relationship between sleep duration and overweight and obesity was assessed using logistic regression models. Both the unadjusted logistic regression model and model adjusted for covariates were applied. Odds ratio (OR) and 95 confidence intervals (95%CI) were computed and expressed in the logistic regression models.

Each NHANES cycle purposely oversampled a certain group of people to increase the representativeness of the sample in the U.S. Sample weight (full sample 2-year MEC examination weight) of the 2015–2016 cycle was applied to all analyses in this study. A *p*-value less than 0.05 was considered significant. All statistical analyses were two-tail test and were performed using SPSS Statistics 20.0 (IBM Corporation. Armonk, NY, USA).

## Results

### Study population

Of the included 2495 participants, 34.39% were Non-Hispanic White, 27.37% were Hispanic, 20.96% were Non-Hispanic Black, 13.51% were Non-Hispanic Asian, and 3.77% were of ethnicities. The median age (Q1, Q3) was 44.00 (31.00–58.00) years old. As of marital status, 63.25% were married or living with a partner, 20.72% were never married, 11.26% were divorced or separated, and 4.77% were widowed. The highest proportion of the education level was college graduate or above (33.23%), followed by some college (31.10%), high school (19.40%), and less than high school (16.27%). PIR groups corresponded to 33.31, 33.19, and 33.51% of the population from the low PIR group to the high PIR group. Most participants did not report trouble in sleep (75.67%). A total of 751 (30.10%) participants were normal weight, 823 (33.00%) were classified as overweight, and 921 (36.91%) were obese. The average sleep duration of the overall population was 7.74 ± 1.24 h, with 64.73% of the study population slept at a normal duration.

### Demographics

The demographic characteristics of the overweight group and obese group were compared with the normal weight group to address potential covariates, as summarized in Table 1. Age (*P* < 0.001), sleep duration (*P* < 0.001), sex (*P* < 0.001), race (*P* < 0.001), marital status (*P* < 0.001), education level (*P* < 0.001), total number of people in the family (*P* = 0.003), number of children aged ≤5 years (*P* = 0.002), and daytime sleepiness (*P* = 0.016) were significantly different among the three weight groups. The median age of the overweight group was the highest across three weight groups (45.00 years [31.00–59.00 years]), followed by the obese group (44.00 years [32.00–58.00 years]) and the normal weight group (41.00 years [27.00–54.00 years]). The average sleep time of the three groups was 7.84 ± 1.15 h, 7.72 ± 1.38 h, and 7.57 ± 1.48 h of the normal weight, overweight, and obese groups, respectively. Baseline variables of the overweight and obese groups were compared with the normal weight group separately to determine the covariates of each weight group, which were not presented in tables. Comparing to the normal weight group, age, sex, race, marital status, and education level were significantly different in the overweight group, which were adjusted in the following overweight analyses. For the obese group, age, sex, race, marital status, education level, total number of family members, and number of children aged ≤5 years were difference from the normal weight group. Thus, these covariates were adjusted in the later obese analyses.

**Table 1:** Demographic characteristics of study participants according to bodyweight status. (NHANES 2015–2016 cycle)

	Total (***n*** = 2495)	Weight Groups^**a**^			*P*^b^
		Normal weight (***n*** = 751)	Overweight (***n*** = 823)	Obese (***n*** = 921)	
**Age (years)**, **M (Q**_**1**_**, Q**_**3**_**)**^**c**^	44.00(31.00–58.00)	41.00(27.00–54.00)	45.00(31.00–59.00)	44.00(32.00–58.00)	< 0.001
**Average sleep time (hours), Mean ± SD**^**f**^	7.74 ± 1.24	7.84 ± 1.15	7.72 ± 1.38	7.57 ± 1.48	< 0.001
**Sleep duration groups**^**g**^**, n (%)**					< 0.001
Short-sleep	513(20.56)	110(11.61)	174(19.90)	229(19.36)	
Normal-sleep	1615(64.73)	526(78.15)	523(66.61)	566(67.86)	
Long-sleep	367(14.71)	115(10.24)	126(13.49)	126(12.77)	
**Sex, n (%)**^**d**^					< 0.001
Male	1244(49.86)	361(43.25)	474(56.31)	409(48.04)	
Female	1251(50.14)	390(56.75)	349(43.69)	512(51.96)	
**Ethnicity, n (%)**					< 0.001
Hispanic	683(27.37)	123(8.14)	257(14.67)	303(16.46)	
Non-Hispanic White	858(34.39)	283(70.37)	290(68.15)	285(63.35)	
Non-Hispanic Black	523(20.96)	126(8.34)	146(8.54)	251(13.98)	
Non-Hispanic Asian	337(13.51)	197(10.65)	102(5.29)	38(2.03)	
Non-Hispanic Others	94(3.77)	22(2.50)	28(3.35)	44(4.18)	
**Marital status, n (%)**					< 0.001
Never married	517(20.72)	211(24.96)	141(15.06)	165(14.75)	
Married/Living with Partner	1578(63.25)	448(66.43)	543(70.67)	587(69.91)	
Divorced/Separated	281(11.26)	56(5.19)	98(10.61)	127(11.86)	
Widowed	119(4.77)	36(3.42)	41(3.66)	42(3.48)	
**Education level, n (%)**					< 0.001
Less than High School	406(16.27)	103(6.81)	144(9.28)	159(9.40)	
High school	484(19.40)	125(12.62)	166(18.45)	193(20.20)	
Some college	776(31.10)	206(28.20)	228(28.70)	342(38.72)	
College graduate or above	829(33.23)	317(52.37)	285(43.58)	227(31.69)	
**Total number of people in the family, n (%)**					0.003
1	541(21.68)	193(26.13)	181(22.34)	167(18.19)	
2–3	982(39.36)	292(42.71)	335(47.98)	355(42.62)	
≥ 4	972(38.96)	266(31.17)	307(29.68)	399(39.19)	
**Number of children aged ≤ 5 years, n(%)**					0.002
0	1947(78.04)	608(83.83)	660(84.36)	679(76.64)	
1–2	524(21.00)	140(15.90)	156(15.11)	228(22.22)	
≥ 3	24(0.96)	3(0.27)	7(0.53)	14(1.14)	
**Number of children aged 6–17 years, n(%)**					0.184
0	1619(64.89)	516(72.58)	537(70.75)	566(67.12)	
1–2	719(28.82)	199(23.20)	235(25.39)	285(26.83)	
≥ 3	157(6.29)	36(4.22)	51(3.86)	70(6.05)	
**PIR**^**e**^**, n (%)**					0.280
< 1.56	831(33.31)	227(19.16)	268(19.65)	336(23.43)	
3.61–4.56	828(33.19)	251(31.89)	277(30.86)	300(28.74)	
≥ 4.56-	836(33.51)	273(48.96)	278(49.49)	285(47.83)	
**Daytime sleepiness, n (%)**					0.016
Never	456(18.28)	175(16.93)	160(13.19)	121(10.60)	
1 time a month	605(24.25)	196(27.76)	179(22.32)	230(23.00)	
2–4 times a month	872(34.95)	237(33.49)	299(39.75)	336(39.64)	
5–15 times a month	409(16.39)	110(16.62)	140(19.89)	159(19.23)	
16–30 times a month	153(6.13)	33(5.20)	45(4.85)	75(7.53)	
**Trouble sleep, n(%)**					0.349
No	1888(75.67)	596(73.63)	638(73.76)	654(69.89)	
Yes	607(24.33)	155(26.37)	185(26.24)	267(30.11)	

^a^Weight groups: normal weight group BMI 18.5–24.9 kg/m^2^, overweight group BMI 24.9–30 kg/m^2^, and obese group BMI ≥30 kg/m^2^

^b^*P* value Results from Pearson’s chi-square test, t-test and Fisher’s exact test

^c^*M (Q1, Q3)* Median (quartile 1, quartile3)

^d^*N%* Frequency and percentage of the total

^e^*PIR* Poverty income ratio

^f^*Mean ± SD* mean ± standard deviation

^**g**^Sleep duration groups were defined as follow: Short-sleep group < 7 h, Normal-sleep group 7–9 h, Long-sleep group > 9 h

### Sleep duration and obesity

The normal-sleep group was determined to be the reference group of all logistic regression analyses (Table 2). When comparing to the normal-sleep group, the short-sleep group indicated a significantly higher odds of overweight (OR = 1.752, 95%CI: 1.243–2.470, *P* = 0.003), while the overweight incidence of the long-sleep group was not statistically different from the reference (OR = 1.346, 95%CI: 0.826–2.193, *P* = 0.214). After adjusting for age, sex, race, marital status, and education level, the pattern remained consistent, illustrating a significantly higher odds of overweight in the short-sleep group (OR = 1.825, 95%CI: 1.251–2.661, *P* = 0.004). The occurrence of obesity was statistically higher in both the short-sleep (OR = 1.832, 95%CI: 1.215–2.762, *P* = 0.007) and the long-sleep group (OR = 1.370, 95%CI: 1.043–1.800, *P* = 0.027) as compared to the reference in the unadjusted model. The short-sleep group indicated a significantly higher odds of obesity (OR = 1.574, 95%CI: 1.201–2.064, *P* = 0.001) after controlling for age, sex, race, marital status, education level, total number of family members, and number of children aged ≤5 years, while no significant difference was observed in the long-sleep group.

**Table 2:** Multivariate logistic regression models assessing the relationship between sleep duration and bodyweight status

	Overall population					
	Unadjusted model			Adjusted model^a^		
**Sleep duration-Overweight**	**OR**^**b**^	**95%CI**^**c**^	***P***^**d**^	**OR**	**95%CI**	***P***
Normal	*Ref*			*Ref*		
Short	1.752	1.243–2.470	0.003	1.825	1.251–2.661	0.004
Long	1.346	0.826–2.193	0.214	1.399	0.793–2.469	0.227
**Sleep duration-Obese**	**OR**	**95%CI**	***P***	**OR**	**95%CI**	***P***
Normal	*Ref*			*Ref*		
Short	1.832	1.215–2.762	0.007	1.574	1.201–2.064	0.001
Long	1.370	1.043–1.800	0.027	1.320	0.896–1.945	0.160

^a^Adjusted model: Overweight analysis controlled for age, sex, race, marital status, and education level, while obese analysis adjusted for age, sex, race, marital status, education level, total number of family members, and number of children aged ≤5 years

^b^*OR* Odds Ratio

^c^95%CI 95% Confident Interval (min-max)

^d^*P* value: Results from logistic regression models

^f^Sleep duration groups were defined as follow: Short-sleep group < 7 h, Normal-sleep group 7–9 h, Long-sleep group > 9 h

### Subgroup analyses

Subgroup analyses further stratified the population by age and sex. When stratifying by age, 436 participants were classified as the older adult group, and 2059 participants were categorized as the adult group (Table 3). In the adult group, the overweight odds of the short-sleep group were significantly higher than that of the normal-sleep group (OR = 2.094, 95%CI: 1.444–3.038, *P* < 0.001), while the long-sleep group showed no statistical difference as compared to the reference (OR = 1.364, 95%CI: 0.875–2.126, *P* = 0.178). When controlling for sex, race, marital status, and education level, the results were allied with the unadjusted model, with the short-sleep group showing a significant increase in overweight incidence (OR = 1.951, 95%CI: 1.333–2.855, *P* = 0.002). A similar pattern was detected in the obese analysis, with the short-sleep group demonstrating a statistically higher occurrence of obesity in the unadjusted (OR = 1.773, 95%CI: 1.181–2.660, *P* = 0.009) and adjusted model (OR = 1.475, 95%CI: 1.085–2.006, *P* = 0.0013) controlling for age, sex, race, marital status, education level, total number of family members, and number of children aged ≤5 years, while no significance was discovered in the long-sleep group. In contrast, findings of the older adult group were divergent from the adult group, displaying no statistical differences in any of the comparisons among the older adults.

**Table 3:** The relationship between sleep duration and bodyweight status: stratified by age

	Adult group (***n*** = 2059)						Older adult group (***n*** = 436)					
	Unadjusted model			Adjusted model^a^			Unadjusted model			Adjusted model		
**Sleep Duration-Overweight**	**OR**^**b**^	**95%CI**^**c**^	***P*** ^**d**^	**OR**	**95%CI**	***P***	**OR**	**95%CI**	***P***	**OR**	**95%CI**	***P***
Normal	*Ref*			*Ref*			*Ref*			*Ref*		
Short	2.094	1.444–3.038	< 0.001	1.951	1.333–2.855	0.002	1.819	0.599–5.521	0.269	1.737	0.564–5.349	0.312
Long	1.364	0.875–2.126	0.157	1.433	0.833–2.466	0.178	1.647	0.736–3.685	0.206	1.367	0.535–3.494	0.489
**Sleep Duration-Obese**	**OR**	**95%CI**	***P***	**OR**	**95%CI**	***P***	**OR**	**95%CI**	***P***	**OR**	**95%CI**	***P***
Normal	*Ref*			*Ref*			*Ref*			*Ref*		
Short	1.773	1.181–2.660	0.009	1.475	1.085–2.006	0.013	2.377	0.817–6.912	0.104	2.464	0.805–7.541	0.114
Long	1.137	0.823–1.571	0.411	1.223	0.807–1.852	0.342	1.705	0.893–3.258	0.099	1.404	0.547–3.603	0.481

Sleep duration groups for older adult ≥65 years were defined as follow: Short-sleep group < 7 h, Normal-sleep group 7–8 h, Long-sleep group > 9 h

^a^Adjusted model: Overweight analysis controlled for age, sex, race, marital status, and education level, while obese analysis adjusted for age, sex, race, marital status, education level, total number of family members, and number of children aged ≤5 years

^b^*OR* Odds Ratio

^c^*95%CI* 95% Confident Interval (min-max)

^d^*P* value: Results from logistic regression models

^e^Sleep duration groups for adult < 65 years were defined as follow: Short-sleep group < 7 h, Normal-sleep group 7–9 h, Long-sleep group > 9 h

In the sex-stratified analyses (Table 4), there were 1244 males and 1251 females in the subgroup analysis. For females, the short-sleep group (OR = 2.491, 95%CI: 1.281–4.844, *P* = 0.01) illustrated an elevated odds of overweight than that of the normal-sleep group when adjusting for age, race, marital status, and education level in the female population. Conversely, the overweight incidence of the short-sleep group (OR = 1.504, 95%CI: 0.980–2.307, *P* = 0.06) was not statistically different from the odds ratio in the normal-sleep group in the male group. The long-sleep duration was not associated with overweight odds in both the female and male groups.

**Table 4:** The relationship between sleep duration and bodyweight status: stratified by sex

	Female (***n*** = 1251)						Male (***n*** = 1244)					
	Unadjusted model			Adjusted model^a^			Unadjusted model			Adjusted model		
**Sleep Duration-Overweight**	**OR**^**b**^	**95%CI**^**c**^	***P*** ^**d**^	**OR**	**95%CI**	***P***	**OR**	**95%CI**	***P***	**OR**	**95%CI**	***P***
Normal	*Ref*			*Ref*			*Ref*			*Ref*		
Short	2.507	1.382–4.547	0.005	2.491	1.281–4.844	0.010	1.776	1.251–2.521	0.003	1.504	0.980–2.307	0.060
Long	1.368	0.599–3.124	0.431	1.289	0.546–3.043	0.539	1.931	1.023–3.647	0.043	1.506	0.670–3.383	0.298
**Sleep Duration-Obese**	**OR**	**95%CI**	***P***	**OR**	**95%CI**	***P***	**OR**	**95%CI**	***P***	**OR**	**95%CI**	***P***
Normal	*Ref*			*Ref*			*Ref*			*Ref*		
Short	2.464	1.265–4.798	0.011	2.590	1.441–4.656	0.001	1.464	0.970–2.210	0.067	1.030	0.694–1.530	0.882
Long	1.551	1.060–2.270	0.027	1.698	1.055–2.734	0.029	1.112	0.758–1.631	0.565	0.815	0.435–1.527	0.523

^a^ Adjusted model: Overweight analysis controlled for age, sex, race, marital status, and education level, while obese analysis adjusted for age, sex, race, marital status, education level, total number of family members, and number of children aged ≤5 years

^b^*OR* Odds Ratio

^c^*95%CI* 95% Confident Interval (min-max)

^d^*P* value: Results from logistic regression models

^e^Sleep duration groups were defined as follow: Short-sleep group < 7 h, Normal-sleep group 7–9 h, Long-sleep group > 9 h

The obesity incidence was significantly higher in the short-sleep group (OR = 2.590, 95%CI: 1.441–4.656, *P* < 0.001), as well as the long-sleep group (OR = 1.698, 95%CI: 1.055–2.734, *P* = 0.029) when controlling for age, sex, race, marital status, education level, total number of family members, and number of children aged ≤5 years. Nevertheless, no association was detected in the short-sleep and long-sleep group of the male analysis.

## Discussion

In this cross-sectional study, we explored the relationship between short sleep time and weight status using the NHANES 2015–2016 database. Our study found that short sleep duration was associated with the incidence of overweight and obesity. Participants who slept less than 7 h were 1.83 times more likely to be overweight and 1.57 times more likely to be obese than participants who sleep 7 to 9 h. When sleeping more than 9 h, participants were 1.37 times more likely to be obese than sleep 7–9 h. The average sleep duration displayed a decremental pattern as the weight increases, with normal weight participants showing the longest average sleep time 7.84 ± 1.15 h, followed by overweight people at 7.72 ± 1.38 h, and obese individuals at 7.57 ± 1.48 h.

Demographic comparisons of the current research illustrated that age, sleep duration, sex, race, marital status, education level, total number of people in the family, number of children aged ≤5 years, and daytime sleepiness were significant predictors of the weight status. Previous studies have established that overweight and obesity are more prevalent in women than men due to the physiological discrepancies in adipose distribution and lipid metabolism [7, 21]. Additionally, researchers have discovered short sleep duration is associated with significantly higher odds of obesity in both men and women, but the pattern of changes in obesity odds indicated a disparity in men and women [22]. Hence, we conducted a sex-stratified subgroup analysis to address the potential differences between the male and female population. Aging, associated with changes in the abdominal white adipose tissue and shorter overall sleep duration [23, 24], is another essential factor that requires more careful consideration. In our subgroup analysis, we stratified the adult population by age, separating the elder adult group from the adult group.

In our age-stratified subgroup analyses, sleep duration was associated with overweight and obesity among the adult group, showing approximately 2 folds increase in the overweight incidence and 1.5 folds increase in the obese incidence of the short-sleep group as compared with the normal-sleep group. Notably, such association was not established among older adults. One possible reason is that aging is accompanied by sensory function decline, including blunt olfactory function and taste, which affect the dietary consumption of older adults since eating is a sensuous activity [25]. Moreover, the increased risk of diseases and complications in older adults may lead to inflammatory responses, impacting the dietary intake and weight [26]. Therefore, the effect of the short-sleep duration on food intake and weight gain may be influenced among older adults.

When stratifying by sex, no association was discovered between sleep duration and overweight and obesity among males. In the female population, 2.49 times higher incidence of overweight was observed in the short-sleep group than the reference, but no association was detected in the long-sleep group. Interestingly, the obesity prevalence was significantly higher in both the short-sleep group and long-sleep group among the female population, corresponding to 2.59 times and 1.70 times higher odds when compared to the normal-sleep group.

Among children and adolescents, a robust relationship between short sleep duration and weight has been elucidated in previous studies [11, 27–31]. However, studies that investigated the adult population uncovered inconclusive results. Allied with our findings, Bonanno et al. revealed an inverse association between sleep duration and BMI among adults [32]. Similarly, Watanabe et al. conducted a cross-sectional analysis of the Japanese population, which revealed a significant relationship between short-sleep time of fewer than 7 h and BMI compared to sleep duration at 7–8 h [33]. Conversely, the Whitehall II study of the British population unearthed an insignificant relationship between BMI and short sleep duration in the prospective analysis [34].

An older NHANES-I study conducted two types of analyses, cross-sectional and longitudinal, to examine inadequate sleep in relation to obesity [35]. The cross-sectional analysis of the NHANES-I study stratified age by 10-year increments. An inverse association was observed between sleep duration and BMI, which is comparable to our results. Nevertheless, the cohort analysis did not reveal a significant relationship between obesity incidence and sleep duration. This NHANES-I study used data from older cycles, 1982–1984, 1987, and 1992, whereas our study used the database of the NHANES 2015–2016 cycle. Moreover, the bodyweight measures of the participants in the longitudinal study were self-reported, decreasing the accuracy of the estimation.

Our results of the older adult group disagree with the previous study on the middle-aged and older adult population. The prospective study conducted by Xiao et al. investigated the National Institutes of Health-AARP (NIH-AARP) Diet and Health Study, illustrating a significant inverse relationship between sleep duration and body weight among older adults [22]. The sample size is one possible explanation of the disparities between our results and the NIH-AARP study. Our study included 436 participants in the older adult analysis, while the final sample of the NIH-AARP study consisted of 43,176 participants. Additionally, the NIH-AARP study assessed body weight through a self-reported questionnaire, while our NHANES study measured body weight during the in-person physical examination by trained medical professionals.

The association between sleep and weight status may be attributed to several factors. Although the exact pathophysiology has not been fully uncovered, various underlying mechanisms have been proposed. Under sleep deprivation, the secretion of satiety regulatory hormones is impaired. Leptin, a hormone secreted by the adipocytes to suppress hunger, is found to decrease significantly during sleep restriction [36, 37]. The long-term reduced hunger-suppressing hormone may result in increased caloric intake and weight gain. From a behavioral perspective, sustained wakefulness allows more time and possibility for food intake, and therefore potentially increases the risk of weight gain.

This research has analyzed nationally representative data from the NHANES database, but some limitations need to be addressed. Since our study is observational, a causal relationship cannot be established. A previous study suggested that obesity may precede inadequate sleep, implying a potential bidirectional association between sleep duration and bodyweight [27]. Moreover, although the sleep questionnaire designed by the NHANES 2015–2016 cycle has considered the habitual and incoherent sleep time [12], certain aspects have not been encompassed. The assessment of sleep duration relied on self-reported information. Participants may report the total time on bed, incorporating sleep latency, rather than the actual sleep duration, which generates deviation of the assessment of sleep duration. Additionally, the timing of sleep, which is not recorded in the NHANES database, has also been linked with weight gain [38]. Therefore, collecting more detailed information regarding sleep duration and time may be implemented in future studies. Furthermore, people with certain physiological characteristics or health conditions may require a different sleep time than the recommendation to achieve protective or beneficial effects. Thus, future studies targeting various population may analyze the sleep durations that best fulfill the need of each distinct population.

## Conclusion

Our findings consolidate the relationship between sleep duration and overweight and obesity. Short sleep duration, less than 7 h, is associated with a 1.83 times higher overweight rate and a 1.57 times higher obesity rate. Long-sleep duration, greater than 9 h, is also associated higher odds of obesity. After stratifying by age, short-sleep duration is associated with 1.95 times higher odds of overweight and 1.48 higher obesity occurrence among adults younger than 65 years old. Similarly, no association is detected in the long-sleep group. Furthermore, the incidence of overweight increased 2.49 times in the short-sleep group of the female population. Short-sleep duration and long-sleep duration are associated with 2.59- and 1.70-times higher prevalence of obesity of the female population. Sleep duration is not related to weight status in the male population. The findings of this research may be used to support clinical recommendations for weight management. Moreover, the disparities observed between the adult and older adult population imply the influence of sleep on body weight may be diminished by aging. When examining the relationship between sleep and weight, future studies on older adults need to consider other physiological changes, as well as potential complications of the elderly population.

## Data Availability

The datasets used and analyzed during the current study are available from the corresponding author on reasonable request.

## Abbreviation

WHO: World Health Organization
BMI: Body Mass Index
NHANES: National Health and Nutrition Examination Survey
MEC: Mobile Examination Center
SLQ: Sleep disorders questionnaire
Q: Quartiles
OR: Odds Ratio
95%CI: 95% Confidence Intervals
OW/OB: Overweight and Obese
PIR: Poverty Income Ratio
NIH-AARP: National Institutes of Health -AARP
